# Enhancement of Near-Field Heat Transfer Performance of a Piezoelectric Synthetic Jet with Outlet Flaps

**DOI:** 10.3390/mi17040440

**Published:** 2026-04-01

**Authors:** Xincai Liu, Yi Hu, Jincheng Hu, Wenjuan Liu, Yuhan Wang, Qi Liu

**Affiliations:** 1 The Institute of Technological Sciences, Wuhan University, Wuhan 430072, China; 2 School of Power and Mechanical Engineering, Wuhan University, Wuhan 430072, China; 3 School of Integrated Circuits, Wuhan University, Wuhan 430072, China; 4 China Ship Development and Design Center, Wuhan 430064, China

**Keywords:** synthetic jet, impingement cooling, flaps, thermal management, heat transfer

## Abstract

This study experimentally investigates a compact side-exhaust piezoelectric synthetic jet actuator equipped with outlet flaps and compares its performance with a flap-free baseline design. The flap concept is intended to mitigate hot-air recirculation during the suction phase and thereby improve near-field cooling in confined layouts. Experiments were conducted under a 350 Hz, 60 Vpp driving signal with an exit dimension of 20 mm × 1 mm. An initial screening campaign evaluated 24 flap configurations by varying flap length, thickness, and installation distance; the results showed that overly long flaps impose substantial blockage and momentum loss, and therefore the flow analysis was narrowed to a practical flap length of 29.5 mm. The final velocity characterization focuses on two representative flap thicknesses (0.1 mm and 0.5 mm) and three installation distances (5, 10, and 15 mm from the exit). For heat transfer evaluation, the nozzle-to-target spacing was varied from 5 to 50 mm in 5 mm increments. The modified actuator demonstrates improved near-field cooling performance, with the best case achieved using 0.1 mm flaps installed at 5 mm, yielding a maximum Nusselt number enhancement of 6.24% relative to the baseline at very small spacings. Furthermore, the thermal benefit becomes more pronounced at elevated heat source temperatures, with the strongest improvement observed around 60–80 °C (up to ~13% at 60 °C). These results provide practical design guidance for enhancing localized convective heat transfer in compact electronics cooling applications.

## 1. Introduction

Advances in technology have led to the widespread use of electronic devices in daily life, accompanied by a continuous increase in their component density. This trend has resulted in a significant rise in heat generation per unit volume [1], as exemplified by applications such as servers [2] and smart wearable devices [3]. Consequently, thermal management has become a critical bottleneck limiting further progress in electronics [4]. Existing cooling techniques include air cooling, liquid cooling [5], heat sink cooling [6] and impinging jet cooling [7,8,9]. Among these, impinging jet cooling is recognized for its exceptionally high heat transfer efficiency, with synthetic jet technology representing a prominent research focus within this field [10].

The synthetic jet technique was first introduced by Smith and Glezer, who primarily investigated its fundamental flow development [11]. Since then, it has found broad applications in areas such as flow control [12,13] and bio-inspired systems [14]. In thermal management research, synthetic jets have been extensively studied. Mahalingam et al. [15] reported substantial heat transfer enhancement under specific baseline conditions (e.g., compared with conventional flows in their configurations). Dutta et al. [16] combined synthetic jets with finned surfaces, achieving significantly improved heat transfer, with optimal performance at 50 Hz and 6 Vpp. Arik et al. [17] observed that heater size considerably affects cooling performance; efficiency notably declines with larger heated areas, limiting the technique’s applicability to large-scale components. Chaudhari et al. [18] integrated synthetic jets with heat sinks, demonstrating a fourfold increase in the heat transfer coefficient. Valiorgue et al. [19] identified a critical stroke length below which the stroke duration strongly influences thermal transport. Lindstrom et al. [20] examined jet evolution through rectangular, trapezoidal, and triangular orifices, providing useful insights for flow control and heat dissipation designs. Dong et al. [21] further combined synthetic jets with nanofluids, finding that Al_2_O_3_ nanoparticles yielded the best heat transfer enhancement. However, a major challenge in applying synthetic jet cooling arises under spatial constraints that require the jet device to be placed close to the heat source. In such configurations, reheating of the working fluid due to flow recirculation can significantly reduce cooling efficiency [22,23].

Prior studies have pursued several passive strategies to alleviate recirculation in confined spaces, including (i) tailoring orifice geometry (e.g., rectangular, elliptical, or rhombic openings) to shape the jet and delay entrainment [23,24,25,26], (ii) employing porous or multi-orifice outlets to promote fresh-air replenishment at very small spacings [27,28], and (iii) introducing biasing elements such as fluid diodes, hybrid jets, or outlet flaps to hinder hot-flow return during suction [29,30,31]. Among these, outlet flaps are particularly appealing for engineering applications because they are simple, lightweight, and do not require additional actuators or external plumbing.

Despite these efforts, design guidance for miniaturized, side-exhaust synthetic jet actuators operating in the near field remains limited, especially regarding how flap geometry and placement trade off jet momentum, recirculation suppression, and cooling performance. Motivated by compact electronics where a side-exhaust layout is often preferred for routing and clearance, this work experimentally evaluates a piezoelectric synthetic jet with outlet flaps at a scale approximately one-eighth of the device reported by Mirikar et al. [31]. The main contributions are:

(1) A compact side-exhaust actuator is developed and integrated with outlet flaps as a passive near-field recirculation control element.
(2) Velocity screening quantifies the effects of flap thickness, length, and installation distance on centerline velocity decay.
(3) Impingement cooling tests across nozzle-to-target spacings and heat source temperatures identify practical design windows for confined thermal management.

The remainder of this paper describes the experimental setup and measurement methods, reports the flow and heat transfer results for representative flap configurations, and discusses implications for compact electronics cooling designs.

## 2. Methods

### 2.1. Experimental Setup

**Figure 1:**
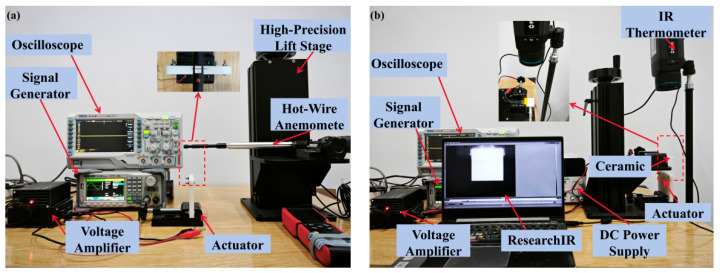
Experimental setup and measurement instruments. (**a**) the configuration for velocity measurements; (**b**) the apparatus for temperature measurements.

Figure 1 illustrates the experimental setup used in this study. Figure 1a shows the configuration for velocity measurements, and Figure 1b depicts the apparatus for temperature measurements. The equipment consists of a signal generator (SDG1022X, Shenzhen, China), an oscilloscope (DS1102Z-E, Suzhou, China), a voltage amplifier (YK-HA190, Jinan, China), a hot-wire anemometer (UT362H, Dongguan, China), a computer, a DC power supply (GPS3010D, Shenzhen, China), an online infrared thermometer (FLIR A600-Series, Wilsonville, OR, USA), a ceramic heating element (MCH alumina, Wuhan, China), and a high-precision lifting frame. All experiments were conducted at an ambient temperature of 20 °C ± 0.5 °C, monitored using a high-precision thermometer (TH602F, Wuhan, China).

**Figure 2:**
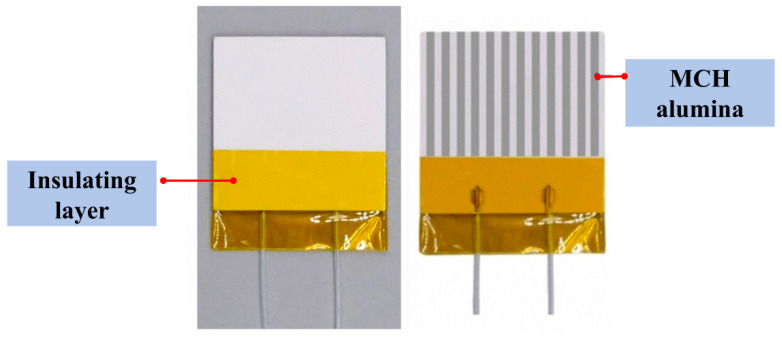
MCH alumina.

The driving unit included the signal generator, oscilloscope, and voltage amplifier. A sine wave signal generated by the signal generator was amplified by the voltage amplifier and monitored on the oscilloscope. After verifying the waveform and amplified voltage, the signal was transmitted to the test apparatus. The oscilloscope provided built-in signals and self-test functions, ensuring stable and reliable input throughout the experiments. Velocity measurements were primarily conducted using a hot-wire anemometer, which utilizes a high-precision platinum resistance thermometer and provides a measurement accuracy of ±4%. Prior to each test, the hot-wire probe was placed inside an enclosed housing. The baseline reading was required to stabilize below 0.02 m/s; otherwise, a standard zero adjustment was performed. Once stabilized, the housing was removed to begin the measurement procedure. Each measurement was repeated six times, and the average value was used for analysis. To assess the reliability of the velocity measurements, each test condition was measured three times. The deviation of each individual measurement from the corresponding mean value was generally within ±0.1 m/s. Therefore, the uncertainty associated with the repeatability of the velocity measurement was estimated to be within ±0.1 m/s for most cases. Temperature measurements were conducted using an online infrared thermometer (FLIR A600-Series), with real-time data displayed and recorded via the ResearchIR 4.40.11 software. The heat source was an MCH alumina ceramic plate (40 mm × 40 mm × 2 mm), as shown in Figure 2, which provided uniform surface heating when powered by a constant voltage from a DC power supply. The infrared thermometer software was monitored until the heat source temperature stabilized, at which point the temperature data were saved. The corresponding heating power indicated on the DC power supply was recorded simultaneously. Multiple trials were performed, and the averaged temperature readings exhibited fluctuations within ±0.1 °C.

**Figure 3:**
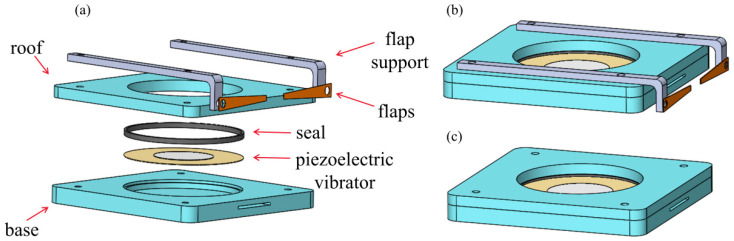
(**a**) The exploded view of the side-exiting piezoelectric synthetic jet actuator. (**b**) The assembled configurations of the actuator with flaps. (**c**) The assembled configurations of the actuator without flaps.

**Figure 4:**
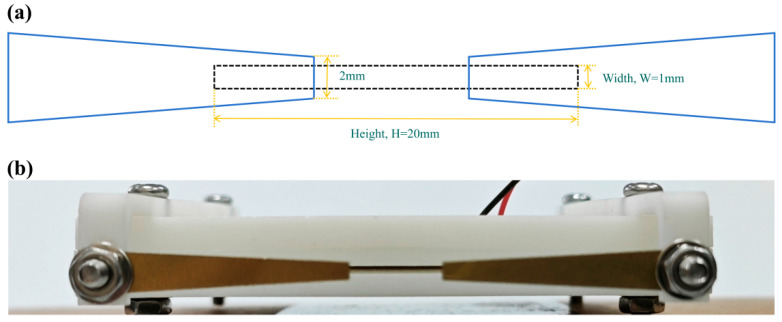
SJ Orifice and flaps viewed from the side view. (**a**) the position of the flaps; (**b**) the fabricated prototype.

Figure 3a shows an exploded view of the side-exiting piezoelectric synthetic jet actuator equipped with outlet flaps. The actuator consists of a base, a piezoelectric vibrator, seal, a roof, a flap support, and the flaps. Both the jet outlet and the inlet channels are located on the side of the base; together, the piezoelectric vibrator and the base form an internal cavity. Key structural parameters are listed in Table 1. The assembled configurations of the actuator with flaps and without flaps are presented in Figure 3b,c, respectively. In the following sections, the flap-equipped design (Figure 3b) is referred to as the “modified actuator,” whereas the flap-free design (Figure 3c) is termed the “baseline actuator”. Figure 4a illustrates the position of the flaps, which are located on both sides upstream of the outlet. These flaps are designed to deflect and disperse the recirculating hot air. Figure 4b presents the fabricated prototype, the main components of which were manufactured using high-precision 3D printing.

### 2.2. Experimental Principle

**Figure 5:**
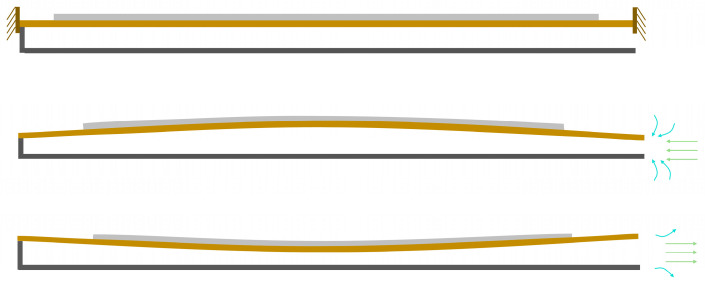
The operating principle of the baseline actuator.

**Figure 6:**
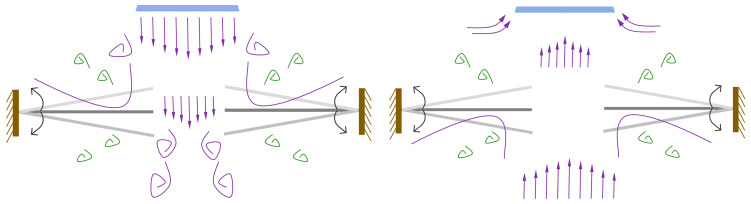
The operating principle of the modified actuator.

Figure 5 illustrates the operating principle of the baseline actuator. During the inhalation phase, the upward expansion of the piezoelectric vibrator draws ambient air into the cavity through the inlet channel. In the subsequent exhalation phase, the downward contraction of the vibrator expels the air through the outlet channel, generating a synthetic jet. Figure 6 depicts the operating principle of the modified actuator, in which both inhalation and exhalation consist of two distinct stages. During inhalation, as the incoming flow approaches the gap between the two flaps, part of the airflow is obstructed and diverted laterally by the flap surfaces. Meanwhile, the central portion of the flow passes through the inter-flap gap, where it is disturbed and redirected toward the inlet channel. In the exhalation phase, when the emerging jet reaches the flap gap, a fraction of the flow is again blocked by the flaps and diffuses outward, while the core flow undergoes a second stage of diffusion after traversing the gap.

**Figure 7:**
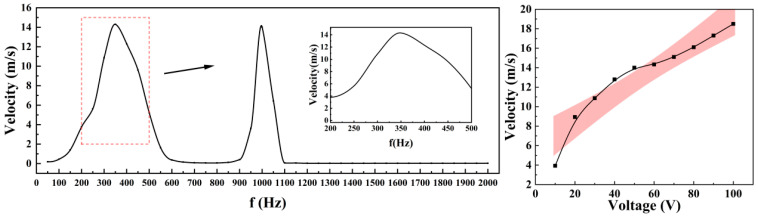
The driving parameters.

The piezoelectric vibrator consists of a piezoelectric ceramic (PZT-5H, Φ40 × 0.2 mm) bonded to an elastic metal plate (beryllium copper, Φ50 × 0.2 mm). The driving parameters were determined experimentally, as summarized in Figure 7. This study investigates the effects of drive frequency on the output speed of prototype components under a constant voltage of 60 Vpp, as well as the influence of drive voltage on output speed at a fixed frequency of 350 Hz. The vibrator operates effectively only within a specific frequency range; outside this range, it cannot generate sufficient oscillation to form a stable jet. Based on these results, a sinusoidal driving signal at 350 Hz and 60 Vpp was selected for all subsequent experiments.

### 2.3. Conditions for Synthetic Jet Formation

The conditions for synthetic jet formation are theoretically determined based on established mathematical models and can be expressed by the following criteria [32]:

(1) $$\frac{1}{S_{t}}=\frac{Re}{S^{2}}>K$$

In the above equation, *S_t_* is the Strouhal number, *Re* is the Reynolds number, and *S* denotes the Stokes number. The constant K equals 1 for two-dimensional configurations and 0.16 for three-dimensional axisymmetric flows.

The dimensionless stroke length *L* is a key parameter for assessing the formation of a synthetic jet and is defined as

(2) $$L=\frac{L_{c}}{D_{h}}$$

In the above equation, *D_h_* represents the jet orifice diameter, and *L_c_* is the stroke length, defined as the distance traveled by the expelled fluid column during one full actuation cycle of the synthetic jet.

The mean outlet velocity of the synthetic jet is expressed as

(3) $$\bar{U}=\frac{L_{c}}{T}$$

Based on the mean outlet velocity, the Reynolds number can be defined as

(4) $$Re=\frac{\bar{U}D_{h}}{v}$$

Therefore, it can be derived that

(5) L=LcDh=U0¯TDh=U0¯fDh=U0¯Dh/vfDh2/v=ReS2=1St

For outlets with a rectangular cross-section, the characteristic dimension of the jet orifice is represented by the hydraulic diameter, which is defined as [33]

(6) $$D_{h}=\frac{2\cdot W\cdot H}{W+H}$$

In the equation above, *W* and *H* represent the shorter and longer sides of the rectangle, respectively. For long, narrow rectangular openings where *H* ≫ *W*, the hydraulic diameter simplifies to *D_h_* ≈ 2 W.

Based on the preceding analysis, a stable and effective synthetic jet can be generated with a fixed actuator geometry, provided that the driving amplitude is sufficient.

### 2.4. Determination of Nusselt Number

To quantitatively evaluate the cooling performance of the actuator, the convective heat transfer coefficient and the corresponding Nusselt number were determined from the measured heater temperature and electrical input power. In the present study, the reported average Nusselt number refers to the area-averaged Nusselt number over the heated surface under steady-state conditions.

The electrical heating power was obtained from the DC power supply and can be expressed as Q = UI. The average convective heat transfer coefficient was then estimated as

(7) $$h=\frac{Q}{A\left(T_{w}-T_{\infty }\right)}$$

where A is the effective heat transfer area of the heater surface, Tw is the area-averaged surface temperature measured by the infrared camera, and $T_{\infty }$ is the ambient air temperature. Based on the calculated heat transfer coefficient, the average Nusselt number was determined by

(8) $$Nu=\frac{hL}{\lambda }$$

where L is the hydraulic diameter of the rectangular outlet and λ is the thermal conductivity of air evaluated under the present test conditions.

For each thermal test condition, three repeated measurements were conducted, and the average values were used for analysis. The 95% confidence interval of the calculated Nusselt number was evaluated from the repeated results according to

(9) $$CI_{95\%}=Nu_{\mathrm{avg}}\pm t_{0.975,n-1}\frac{s}{\sqrt{n}}$$

where *Nu_avg_* is the mean Nusselt number, s is the sample standard deviation, and n is the number of repeated measurements. Since the repeated surface-temperature variation was typically within 0.1 °C, the resulting confidence intervals of the calculated Nusselt numbers were relatively narrow for all tested cases.

For each thermal test condition, three repeated measurements were conducted, and the 95% confidence interval of the average Nusselt number was evaluated from the repeated results. Since the repeated surface-temperature variation was typically within 0.1 °C, the resulting confidence intervals of the calculated Nusselt numbers were relatively narrow for all tested cases.

## 3. Results

This section describes the experimental characterization of the flow and heat transfer performance for both the modified and baseline actuators. For the flow characterization, three geometric parameters of the outlet flap length (29.5 mm, 32 mm), thickness (0.1 mm, 0.2 mm, 0.5 mm), and installation distance from the jet outlet (5 mm, 7.5 mm, 10 mm, 15 mm) were initially considered. A total of 24 flap configurations were tested in the preliminary screening campaign.

The preliminary results indicated that excessive flap length introduced significant blockage and momentum loss, leading to a pronounced reduction in the jet centerline velocity. Therefore, to improve the information density and focus on practically relevant designs, the flow tests were narrowed to the 29.5 mm flap length. In addition, flap thickness was reduced to two representative values (0.1 mm and 0.5 mm) to capture the thin–thick contrast, and the installation distance set was streamlined by omitting the intermediate 7.5 mm case, since the remaining distances (5, 10, and 15 mm) were sufficient to characterize the near-, intermediate-, and far-installation trends. As a result, the final flow characterization reported in this work includes six representative cases (two thicknesses × three installation distances) at the selected flap length, as listed in Table 2.

In these thermal tests, the separation distance between the heat source and the jet outlet was varied from 5 to 50 mm in 5 mm increments to investigate its effect on the cooling performance of each actuator.

### 3.1. Analysis of the Synthetic Jet Velocity Field

**Figure 8:**
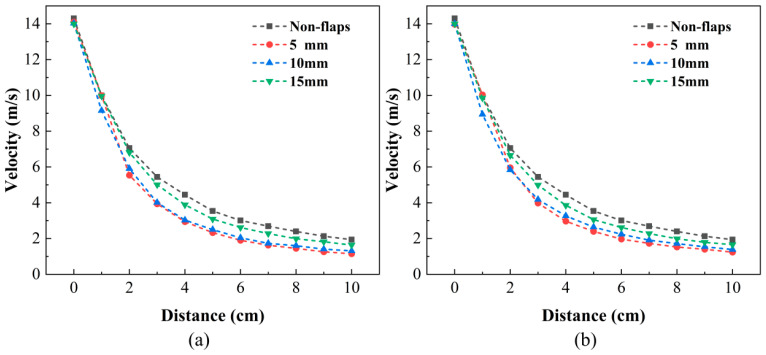
Effect of flap thicknesses on centerline velocity. (**a**) 0.1 mm. (**b**) 0.5 mm.

Figure 8a,b show the decay of the centerline velocity for two different flap thicknesses: 0.1 mm in Figure 8a and 0.5 mm in Figure 8. All cases show a rapid decay in the near field followed by a more gradual decrease downstream, which is characteristic of synthetic jets. Relative to the baseline, the addition of flaps generally reduces the centerline velocity due to exit blockage and additional flow turning. Based on the measured outlet velocity, the Reynolds number was approximately 1.8 × 10^3^ for all tested cases. Since the repeatability-related uncertainty of the velocity measurement was within ±0.1 m/s, the observed trends in Figure 8 were considered reliable under the present experimental conditions.

A strong dependence on the installation distance is observed. When the flaps are mounted close to the outlet, the interaction occurs while the jet is still highly concentrated and momentum-rich, leading to the largest reduction in centerline velocity and a steeper near-field decay. Increasing the installation distance to 10 mm alleviates this penalty, and the centerline velocity becomes closer to the baseline profile. When the flaps are placed at 15 mm, the curves for both flap thicknesses nearly collapse toward the baseline over most of the measured range, indicating that the disturbance introduced by the flaps becomes much weaker when installed farther downstream.

Regarding flap thickness, the 0.1 mm and 0.5 mm curves remain close to each other at each installation distance (Figure 8a,b), suggesting that, for the selected 29.5 mm flap length, thickness has a secondary effect on the maximum centerline velocity compared with installation distance.

**Figure 9:**
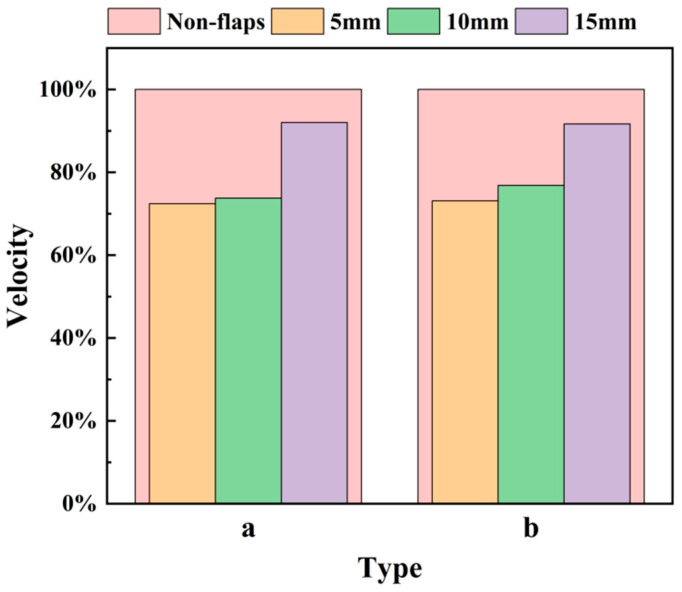
Effect of flap installation position on centerline velocity at 3 cm from the outlet. (**a**) 0.1 mm thickness. (**b**) 0.5 mm thickness.

To provide a compact engineering metric, the velocity ratio at x = 3 cm downstream of the outlet is summarized in Figure 9, where Type a corresponds to the 0.1 mm flap and Type b corresponds to the 0.5 mm flap. The x = 3 cm position is selected as a representative location near the end of the strong near-field decay region, enabling a consistent comparison of the momentum penalty imposed by the flaps.

As shown in Figure 9, the velocity ratio increases monotonically with installation distance for both thicknesses. At 5 mm, the velocity ratio is approximately 0.72–0.74, indicating a reduction of about 26–28% relative to the baseline. At 10 mm, the ratio increases slightly to around 0.74–0.77, corresponding to a smaller velocity penalty than the 5 mm case. At 15 mm, the ratio rises to approximately 0.92–0.93, meaning that the modified actuator retains most of the baseline centerline velocity. Comparing Type a (0.1 mm) and Type b (0.5 mm) at each installation distance shows only minor differences, confirming that installation distance is the dominant parameter governing velocity preservation, while flap thickness plays a limited role in terms of centerline peak velocity.

Overall, the revised flow characterization indicates a practical design trade off: placing flaps closer to the outlet provides stronger flow modulation but incurs a larger momentum penalty, whereas placing flaps farther downstream preserves jet momentum and yields a centerline velocity field closer to the baseline. These results serve as a basis for selecting representative configurations for subsequent thermal performance evaluation under confined, near-field cooling conditions.

### 3.2. Analysis of the Synthetic Jet Temperature Field

#### 3.2.1. The Effect of Heat Source Distance

**Figure 10:**
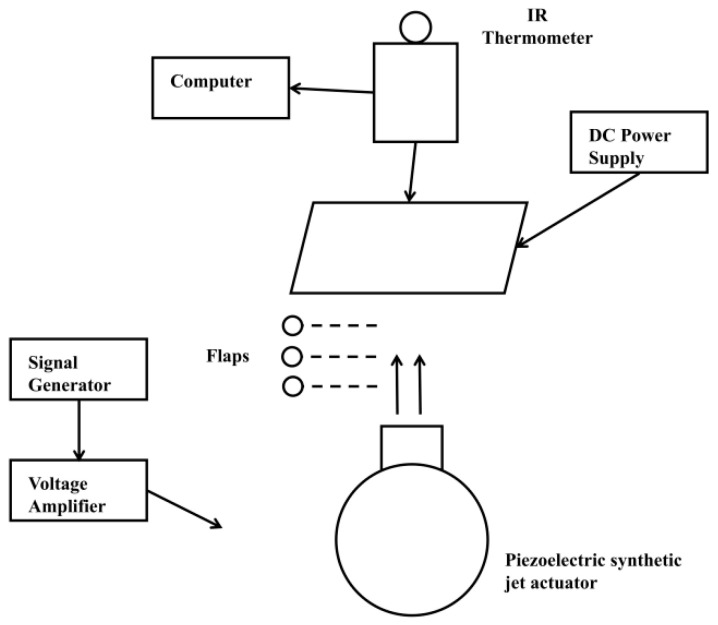
Diagram of the measuring station.

**Figure 11:**
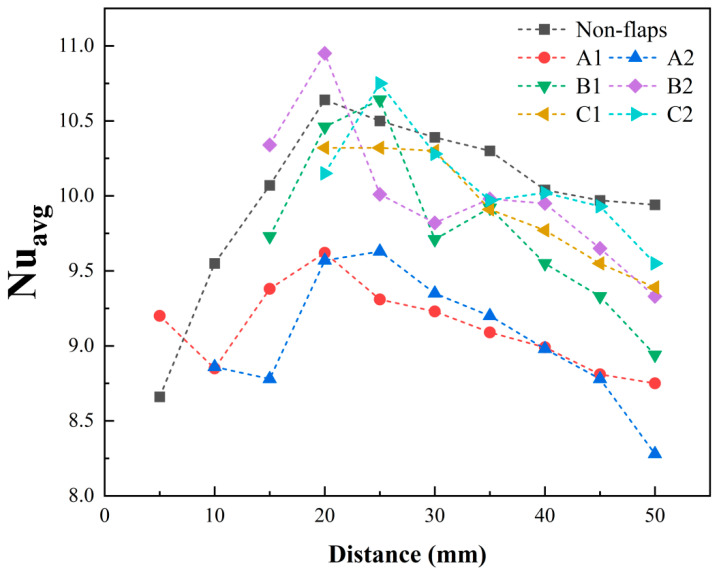
Effect of heat source distance on average Nu: Comparison of 7 device models.

To systematically evaluate the effect of different flap structures on cooling performance, comparative thermal experiments were conducted on six modified actuator configurations (A1, A2, B1, B2, C1, C2) and the baseline prototype. During testing, the heat source was aligned perpendicular to the jet flow direction, as shown in Figure 10. All thermal tests were carried out with a heater input voltage of 6 V. The distance between the heat source and the jet outlet was varied from 5 mm to 50 mm in 5 mm increments. For each distance, the average surface temperature of the heat source was measured. In the present study, the average Nusselt number refers to the steady-state area-averaged Nusselt number over the heated surface. The calculated average Nusselt number are presented in Figure 11. For all tested thermal conditions, the 95% confidence intervals of the calculated Nusselt numbers were evaluated from repeated measurements. For brevity, these values are not listed individually here; however, all of them were found to be smaller than 0.1.

Overall, the average surface temperature and average Nusselt number of the heat source follow similar trends across all configurations. Each model exhibits an optimal cooling range when the heat source is positioned 20–25 mm from the outlet, where heat dissipation is maximized. Compared to the baseline actuator, the modified actuators show distinct performance variations with distance. At a close distance of 5 mm, modification A1 outperformed the baseline, achieving the highest cooling efficiency with a 6.24% increase in Nusselt number. Beyond 10 mm, A-type modifications gradually exhibited weaker cooling than the baseline, with maximum reductions in Nusselt number exceeding 15%. Notably, the cooling curve of the A-type modification displays a distinct peak in the near field, followed by a dip and subsequent recovery, suggesting a shift in its cooling mechanism at certain distances. For the C-type modification, the Nusselt number consistently remained within ±4–5% of the baseline.

**Figure 12:**
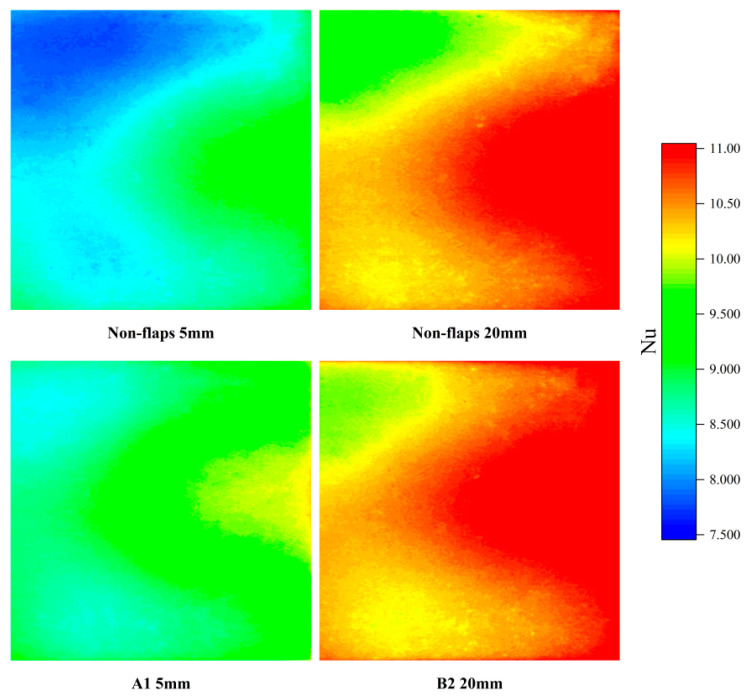
Contour plot of Nu.

The Nusselt number contour plot of the heat source surface further illustrates the performance differences described above. The spatial distribution shown in the contour plot follows the same trend as the average Nusselt number curve. Several representative positions are annotated in the Figure 12.

As shown in Figure 12, at the 5 mm position, the surface Nusselt number distribution differs noticeably between the baseline actuator and modification A1. The A1 configuration exhibits improved heat dissipation across the entire surface, evidenced by the reduction in the large lower-Nu (blue) region in the upper-left portion of the plot. At 20 mm, the high-Nusselt-number area of modification B2 also covers a larger region compared to the baseline. While the overall average Nu increased by only about 4%, the isocontour diagram shows a pronounced expansion of the high-Nu zone in the upper-right section.

#### 3.2.2. The Effect of Heat Source Temperature

**Figure 13:**
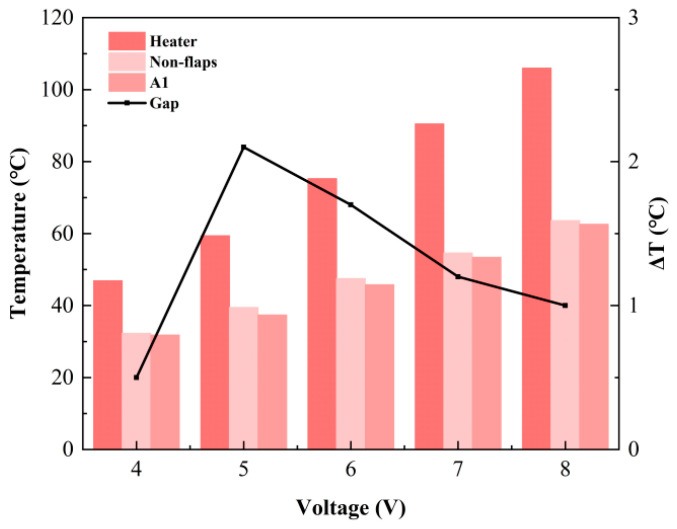
Average temperature on the heater surface at different applied voltages.

**Figure 14:**
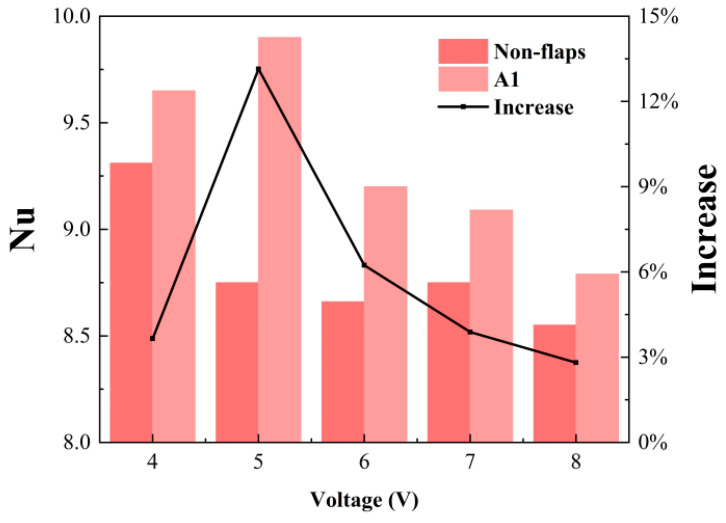
Average Nu on the heater surface at different applied voltages.

To investigate the relationship between cooling performance and heat source temperature, the A1 configuration—identified in previous tests as the best-performing design—was compared with the baseline prototype under varying thermal loads. During the experiments, the DC voltage supplied to the ceramic heater was adjusted to stabilize the heat source at different setpoint temperatures. The relationship between applied voltage and heat source temperature shows a strong positive linear trend, with a correlation coefficient of R^2^ ≈ 0.9987. The corresponding average surface temperature and average Nusselt number of the heat source are presented in Figure 13 and Figure 14.

The results indicate that the cooling efficiency of both devices gradually increases with temperature before stabilizing. Over the applied voltage range of 4–8 V, the cooling efficiency rises from about 30% to approximately 40%, beyond which it plateaus near 40%. Furthermore, the average Nusselt number (Nu) distribution confirms that the A1 modified actuator consistently surpasses the baseline design across all tested temperatures, demonstrating that the flap structure provides a stable heat transfer enhancement. However, the Nu improvement achieved by the A1 modification does not vary monotonically with heat source temperature. It initially increases, then declines, reaching a maximum enhancement of 13% at 60 °C, with a sustained noticeable increase observed across the 60–80 °C range. At relatively low and high temperatures, the improvement remains around 3–4%. Additionally, the Nu trends of the two devices differ. The baseline actuator attains its highest Nu at lower temperatures and remains largely stable elsewhere. In contrast, the A1 modification exhibits an Nu profile that rises to a peak at 60 °C before decreasing.

These results demonstrate that heat source temperature is a critical factor governing the enhanced performance of the flapped actuator. An optimal temperature range (approximately 60–80 °C) exists for maximizing the additional cooling benefit provided by the flaps. Within this range, the A1 configuration most effectively augments the synthetic jet’s cooling capacity.

**Figure 15:**
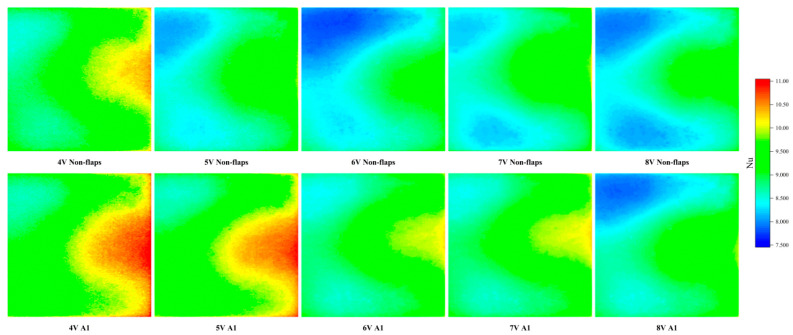
Nu contour plot at different applied voltages.

The Nusselt number contour plots of the heat source surface (shown in the following Figure 15) provide further clarification of these results. The spatial distribution in the contours correlates well with the trend observed in the average Nusselt number maps. Overall, the surface Nu distribution of the A1 modified actuator is markedly improved compared to that of the baseline actuator. This enhancement is also visually discernible in the contour plots. Significant differences in the Nu contours between the modified and baseline configurations emerge at applied voltages of 5 V and 6 V, where the A1 modification exhibits distinct regions of high Nu within the jet-impingement zone.

Furthermore, the contour plots reveal a notable contrast in the distribution of low-Nu regions. For the baseline actuator, pronounced low-Nu zones are present under driving voltages of 5 V, 6 V, 7 V, and 8 V. In contrast, for the A1 modification, distinct low-Nu regions are observed only at 8 V.

## 4. Discussion

### 4.1. Comparison Between Modified Actuator and Baseline Actuator

**Figure 16:**
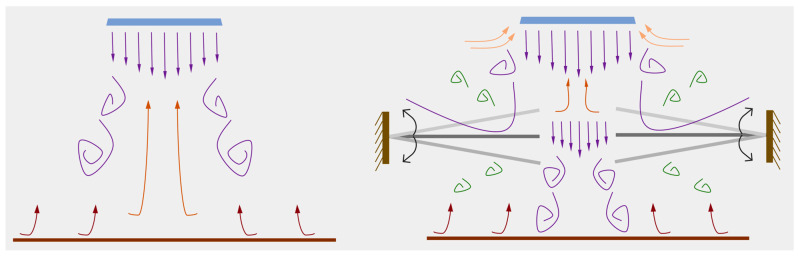
The cooling mechanism of the modified actuator.

Analysis of Figure 16 reveals significant differences in jet behavior between the modified actuator and the baseline actuator, which fundamentally accounts for their divergent thermal dissipation and velocity distribution characteristics.

During the exhalation phase, the baseline actuator produces a typical free jet. The high-velocity core flow impacts the surroundings and entrains adjacent stagnant air, subsequently dispersing outward. In contrast, the introduction of flaps along the jet path in the modified actuator alters the flow structure: when the central jet impinges on the flap surfaces, part of the flow diverges laterally around the flaps. Simultaneously, the flow through the central region is reduced due to the blockage effect of the flanking flaps, thereby decreasing the jet volume between them. This altered flow structure directly affects the centerline velocity distribution. When the jet impinges on the flaps, momentum loss leads to velocity decay. The closer the flaps are to the outlet, the earlier this decay begins, causing the centerline velocity profile to deviate sooner from the free-jet decay trend of the baseline actuator. Conversely, when flaps are positioned farther from the outlet, the jet undergoes a longer period of natural development before impingement. By this stage, the velocity has entered the slowly decaying region of the trajectory, where the flaps exert minimal influence on the overall distribution; consequently, the velocity profile more closely resembles that of the baseline.

**Figure 17:**
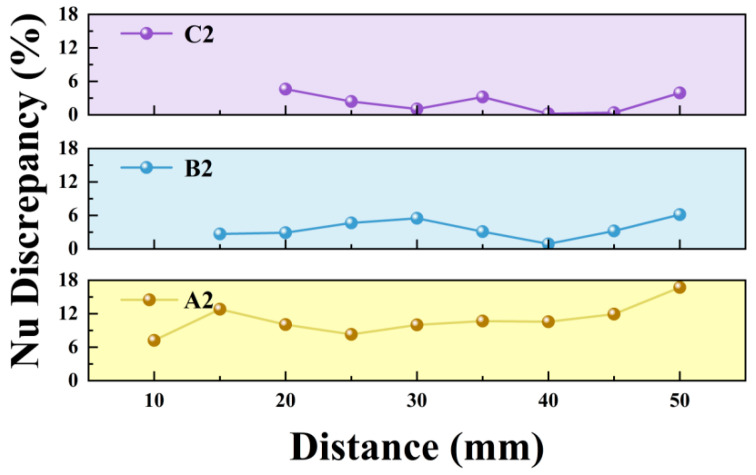
Absolute discrepancy in Nu.

With regard to thermal performance, the effect of the flaps is strongly linked to their installation position. Figure 17 illustrates the absolute difference in Nusselt number between configurations with flaps at various installation distances. It is evident that when flaps are mounted at 10 mm and 15 mm, the difference in cooling capability relative to the baseline is smaller than at other positions, consistent with the aforementioned influence of installation distance on velocity distribution.

### 4.2. Optimal Conditions for Synthetic Jet Cooling with Flaps

**Figure 18:**
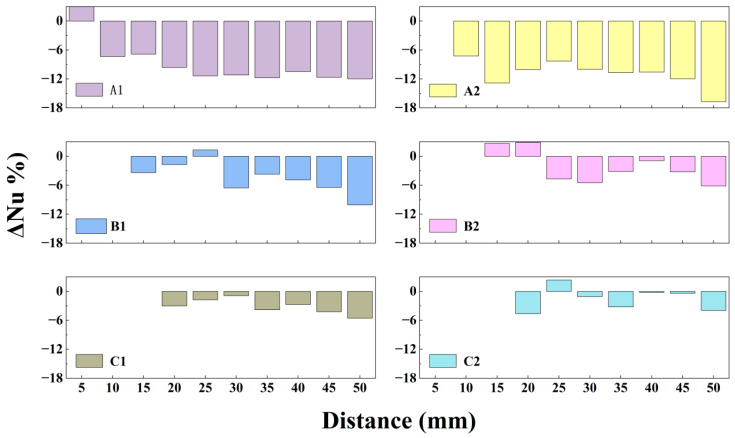
Relative difference in Nu.

When applying synthetic jet devices to practical thermal management scenarios, an optimal heat dissipation distance range exists theoretically; however, complex operational constraints often prevent ideal conditions from being fully realized. As show in Figure 18, for heat sources positioned very close to the jet outlet (e.g., within 5 mm), modified actuators equipped with thin flaps generally deliver superior cooling performance. This advantage arises from the ability of the flap structure to suppress hot-air recirculation effectively. Moreover, the flow disturbance induced by thin flaps in the near-field region moderately disrupts the thermal boundary layer on the heat source surface, thereby enhancing convective heat transfer [34].

As the separation distance between the heat source and the jet outlet increases, B- and C-type actuators installed farther from the outlet achieve thermal performance comparable to or slightly better than that of the baseline actuator. This occurs both at the optimal cooling distance and over a subsequent range. The underlying mechanism can be attributed to two combined effects: the attenuated decay of the jet velocity and the obstruction of hot fluid recirculation by the flaps. Nevertheless, considering the increased structural complexity and manufacturing cost associated with adding flaps, the simpler baseline design remains a more economical and practical choice when the heat source is located farther away.

Furthermore, the heat source temperature itself is a key variable influencing cooling performance. Experimental results show that the near-field cooling advantage of the modified actuator varies notably with temperature. Although the modified design consistently outperforms the baseline across all tested temperatures, the performance gap narrows considerably at lower temperatures. This occurs because hot-air recirculation effects diminish under low-temperature conditions, reducing the flap’s ability to reorganize the flow. Conversely, at excessively high temperatures, the limited size and flow rate of the device constrain the total cool air entrainment, causing the cooling capacity to approach saturation. Consequently, an optimal temperature range exists within which the flap-induced enhancement is maximized, and within this range the performance superiority of the modified actuator is most pronounced.

In summary, practical implementation of synthetic jet-based cooling requires integrated consideration of heat source proximity, operating temperature, and system complexity. For close-range, moderate-temperature applications, lightweight flapped modifications can substantially improve thermal efficiency. For longer-distance scenarios or where structural simplicity is prioritized, the baseline configuration is more suitable. These insights provide a clear theoretical foundation and selection guidance for tailored thermal design under diverse operational requirements.

### 4.3. Factors Affecting Heat Dissipation Efficiency

In practical cooling applications, synthetic jet devices exhibit an optimal range of separation distances from the heat source. When placed too close to the outlet, the jet flow lacks sufficient space to develop before impingement, limiting the effective cooling area. Moreover, it cannot entrain an adequate volume of ambient cool air to form the vortex structures necessary for enhanced heat exchange. Conversely, when the heat source is positioned too far away, the jet velocity decays substantially during propagation, reducing its momentum and consequently weakening its cooling capacity. Therefore, both the baseline and the flap-modified actuators achieve their best thermal performance only within a specific distance interval.

At close separation distances (e.g., within 5 mm), the modified actuator with thin flaps provides significantly higher cooling efficiency. This advantage originates from two main mechanisms. First, the flap structure effectively suppresses the recirculation of heated air; second, the thin flaps may introduce additional unsteadiness in the near-exit flow (e.g., small-amplitude flutter or vibration), which can promote thermal boundary-layer disturbance on the heated surface and enhance convection. As a result, within this near-field range, the modified design demonstrates a clear performance superiority over the baseline.

At larger separation distances, the modified actuator can still maintain cooling performance comparable to or slightly better than that of the baseline. However, given that the addition of flaps increases structural complexity and manufacturing cost—and that the relative cooling gain diminishes with distance—the simpler baseline actuator often represents a more practical and cost-effective solution for far-field applications.

## 5. Conclusions

This study presents a systematic experimental investigation on a side-exiting piezoelectric synthetic jet actuator modified with outlet flaps, aiming to enhance near-field convective cooling in spatially constrained environments. The key findings and engineering implications are summarized as follows:

1. The addition of flaps at the outlet modifies the flow structure at the jet exit. Although this design reduces the jet velocity, it enhances heat transfer primarily by mitigating hot-air recirculation; in addition, thinner flaps may induce stronger unsteady disturbances near the outlet, which can further contribute to boundary-layer disruption and local convection enhancement.
2. Influence of flap parameters: Flap thickness has a negligible effect on the centerline jet velocity. Its impact on heat transfer depends largely on the installation position; at narrow spacings, thin flaps may provide additional heat transfer enhancement by introducing stronger unsteady disturbances in the near-field flow. Flap length significantly influences velocity decay—increasing the projected area accelerates velocity reduction. The installation position of the flaps exhibits the most pronounced influence on both velocity decay and heat transfer. As the installation distance increases, both velocity distribution and cooling efficiency gradually converge toward those of the baseline configuration.
3. Optimal operating conditions: The modified configuration shows superior cooling performance when the heat source is positioned very close to the outlet. Under such conditions, heat dissipation in the baseline design is significantly impaired by hot-air recirculation. The temperature of the heat source also non-monotonically influences the cooling performance of the modified design. An optimal temperature range was identified in this study between 60 and 80 °C. At lower temperatures, hot-air recirculation is less significant; at higher temperatures, the device’s flow rate becomes limiting, and its cooling capacity reaches a plateau.
4. Under compact heat dissipation constraints with moderate heat source temperatures, the modified actuator achieves optimal cooling. It effectively suppresses the recirculation of hot air. When the heat source is located far from the outlet, no significant difference is observed between the modified and baseline actuators. Considering structural simplicity, however, the baseline actuator remains the more practical option in such cases.

In summary, this work demonstrates that strategically designed outlet flaps provide a simple yet effective passive control method to tailor the near-field flow of a synthetic jet, directly addressing the critical challenge of hot-air recirculation in confined spaces. The findings offer clear design guidelines for implementing synthetic jet-based thermal management in next-generation compact electronic systems.

## Figures and Tables

**Table 1:** Parameters.

Type	Value (mm)
Overall size	80 × 80 × 10
Cavity	Φ48 × 2
Outlet	20 × 1
Outlet depth	16

**Table 2:** Test matrix.

Length (mm)	Thickness (mm)	Distance (mm)		
		5	10	15
29.5	0.1	A1	B1	C1
	0.5	A2	B2	C2

## Data Availability

The original contributions presented in this study are included in the article. Further inquiries can be directed to the corresponding author.
